# Sensory Nerve Conduction Velocity Predicts Improvement of Hand Function with Nerve Gliding Exercise Following Carpal Tunnel Release Surgery

**DOI:** 10.3390/jcm10184121

**Published:** 2021-09-13

**Authors:** Yoshiki Tamaru, Akiyoshi Yanagawa, Akiyoshi Matsugi

**Affiliations:** 1 Faculty of Rehabilitation, Shijonawate-Gakuen University, Hojo 5-11-10, Daitou, Osaka 574-0011, Japan; eubrj601@ican.zaq.ne.jp; 2 Department of Rehabilitation, Tesseikai Neurosurgical Hospital, Nakano Honmachi 28-1, Shijonawate, Osaka 575-8511, Japan; akipara@yahoo.co.jp

**Keywords:** carpal tunnel syndrome, nerve gliding exercise, sensory nerve conduction velocity

## Abstract

This study aims to investigate the effects of nerve gliding exercise following carpal tunnel release surgery (NGE-CTRS) and the probing factors affecting the effect of NGE-CTRS on hand function. A total of 86 patients after CTRS participated. Grip strength (grip-s), pinch strength (pinch-s), Semmes-Weinstein monofilament test (SWMT), two-point discrimination (2PD), numbness, pain, and Phalen test (Phalen) were measured and compared between pre- and post-NGE-CTRS. The results showed that the combination of surgery and NGE significantly improved the postoperative grip-s, pinch-s, SWMT, 2PD, numbness, and Phalen; however, no improvement was observed in pain. Background factors that influenced the improved grip-s and pinch-s included gender and preoperative sensory nerve conduction velocity (SCV). Additionally, numbness and Phalen were not affected by age, gender, fault side, bilateral, trigger finger, dialysis, thenar eminence atrophy, motor nerve conduction velocity, SCV, the start of treatment, and occupational therapy intervention. In conclusion, the combination of surgical procedures and NGE showed a high improvement. SCV and time-to-start treatment of intervention for carpal tunnel syndrome may be useful in predicting the function after the intervention.

## 1. Introduction

Carpal tunnel syndrome (CTS) caused by median nerve compression at the wrist is considered the most common entrapment neuropathy [1] with a prevalence of 2–4% in the general population [2,3]. CTS symptoms include pain, paresthesia, numbness, or tingling involving the fingers innervated by the median nerve. Symptoms are worst at night and upon waking up [4]. Patients with severe CTS present with thenar atrophy and loss of sensation [5], which results in gradual weakness and loss of hand function [6,7]. Treatment for CTS with severe symptoms involves surgical procedures to open the carpal tunnel and relieve pressure [8]. Moreover, non-surgical interventions, such as wrist splint [9,10,11,12,13,14], ultrasound [4,15,16,17], steroid injections [18,19,20], and nerve gliding exercise (NGE) [10,11,12,21], were administered for mild to moderate symptoms.

Recently, several systematic reviews have advocated nerve and tendon gliding exercises as a biologically plausible alternative for traditionally advocated treatment modalities in the conservative management for CTS [10,11,12,22,23].

Previous studies on NGE effects have reported improved pinch strength (pinch-s) [24], grip-strength (grip-s) [24,25], and pain [26]. Reports of the effect of no treatment, include pain [26,27], low functional performance [26,27,28], and sensation [15,24,25,27,29]. Thus, the effect of NGE on strength can be expected; however, it does not affect pain, functional performance, or sensation. Since conservative care is ineffective, surgical treatment should be considered. Although surgical treatment can improve symptoms, postoperative care is also important. Degnan et al. (1997) [30] described the importance of controlling edema early postoperatively for CTS. Furthermore, Cook et al. (1995) [31] reported that hand and wrist exercises should be started early in the postoperative period because splinting of the wrist after CTS surgery may cause bowstringing. Steyers et al. (2002) [32] reported that postoperative care should include early mobilization to encourage tendon and nerve gliding. The beneficial effects of these exercises may include direct mobilization of the nerve, facilitation of venous return, edema dispersal, decreased pressure inside the perineurium, and decreased carpal tunnel pressure [10,33]. Thus, it is recommended to surgically decompress the carpal tunnel in patients with severe CTS; however, NGE should be performed as part of the postoperative care.

Therefore, our research hypothesis is that the combination of surgery and NGE for CTS will improve symptoms and that background factors may contribute to the symptom improvement. Thus, this study aims to determine whether the combination of surgery and NGE improves CTS symptoms and what background factors affect the symptom improvement.

## 2. Materials and Methods

### 2.1. Participants

A total of 86 outpatients (31 men and 55 women; mean age 66.8 ± 14.1 years) participated in this study. Table 1 shows the patient characteristics. The inclusion category comprised of those who were prescribed occupational therapy after carpal tunnel release surgery. Exclusion criteria included those who were diagnosed with complex regional pain syndrome, difficulty with NGE due to severe pain, and difficulty understanding instructions.

All participants were informed of the aim of the study and were requested to provide signed informed consent before participation. This study was approved by the Shijonawate-Gakuen University of Faculty of Rehabilitation research ethics review committee (approval no. 21-2) and conducted in accordance with the Declaration of Helsinki.

### 2.2. NGE

**Figure 1:**
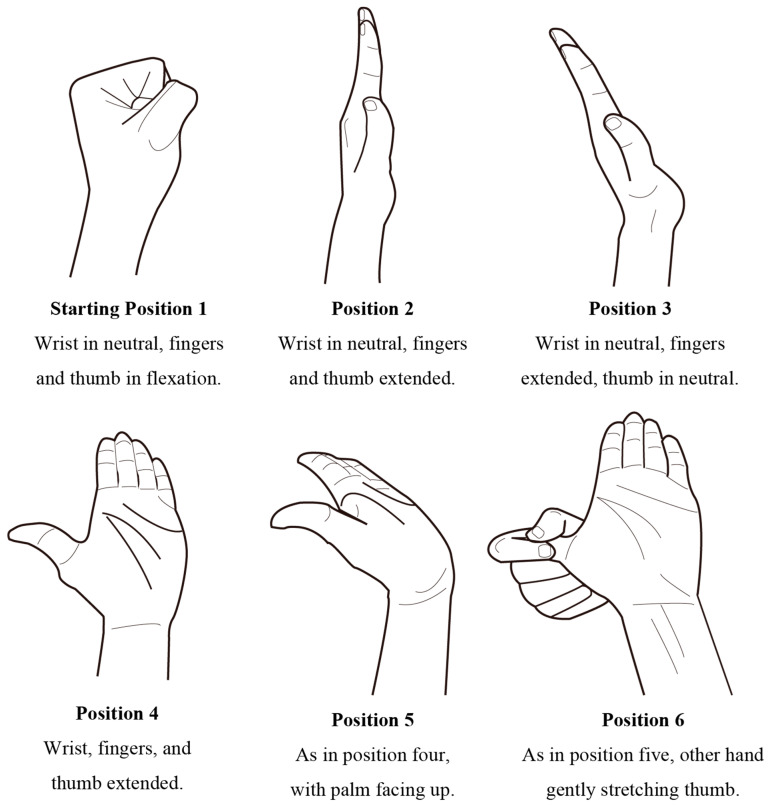
Nerve gliding exercise has six movements depending on the position of the wrist joint and fingers [34].

This study used the NGE method by Nazarieh et al. [34]. Procedures for conducting an NGE (position 1: wrist in neutral and fingers and thumb in flexion; position 2: wrist in neutral and fingers and thumb extended; position 3: thumb in neutral and wrist and fingers extended; position 4: wrist, fingers, and thumb extended: position 5, the same as position 4 with the forearm in supination (palm up); and position 6: same as position 5 with the other hand gently stretching the thumb) were observed (Figure 1).

The NGE was started 4 days postop and performed three times a day during outpatient treatment and self-exercise at home. The NGE is performed in six positions, holding each position for 7 s. These were performed for five sets [35].

### 2.3. Outcome Measures

Basic information was collected on age, gender, fault side, with/without surgery on both sides (bilateral), with/without trigger finger (trigger finger) [11,15], with/without dialysis (dialysis), with/without thenar eminence atrophy (TEA) [11,15,36], preoperative motor nerve conduction velocity (MCS), and preoperative sensory nerve conduction velocity (SCV), time-to-start treatment (start-treat), and occupational therapy intervention period (OT-inter). Additionally, grip-strength (grip-s), pinch strength (pinch-s), Semmes-Weinstein Monofilament test (SWMT), two-point discrimination (2PD), numerical rating scale score for numbness, numerical rating scale score for pain (pain), and positive angle on Phalen test (Phalen). Each test was measured at a frequency of 1 week postoperatively.

#### 2.3.1. Grip-s and Pinch-s

CTS reduces grip-s and pinch-s [37], which were evaluated with the use of dynamometry [5,15,24].

#### 2.3.2. Semmes-Weinstein Monofilament Test (SWMT)

The SWMT is a noninvasive sensory testing method. Monofilaments of different thicknesses are applied to the test area for 1 s. Scoring is determined according to the thickness of the monofilament perceived to be touched [12,38,39].

#### 2.3.3. Two-Point Discrimination (2PD)

A measure of sensory acuity and light touch—2PD—is tested by measuring the smallest distance in a patient to perceive two pinpricks as separate units. This is a commonly used method for assessing the sensory function of the median nerve [15,24,36].

#### 2.3.4. Numerical Rating Scale (NRS) for Numbness and Pain

The NRS has a numerical range from 0 to 10, with 0 indicating nothing at all and 10 indicating the worst. In this study, we assessed the degree of pain and numbness according to the NRS [40].

### 2.4. Statistical Analysis

Paired Student’s *t*-test was used to compare whether the combination of CTS surgery and NGE improves grip-s (kgf), pinch-s (kgf), SWMT, 2PD (mm), numbness (NRS), pain (NRS), and Phalen test (angle) at pre- and post-intervention. This test can reveal the effect of CTS surgery and NGE on these parameters that reflect hand function. Multiple regression analysis was performed with endpoints of the effect of combined surgery and NGE as dependent variables and fault side, gender, age, trigger finger, dialysis, bilateral, TEA, MCS, SCV, Start-treat, and OT-inter as independent variables to determine which of the basic characteristics (age, gender, fault side, bilateral, trigger finger, dialysis, TEA, MCV, SCV, start-treat, and OT-inter) affect the parameters associated with hand function at the end of the treatment. The alpha level was set at 0.05.

## 3. Results

### 3.1. Comparison between Preintervention and Final Assessments

Comparison between the two groups of preoperative and final evaluations showed significant improvement in grip-s (*p* = 0.04), pinch-s (*p* = 0.007), SWMT (*p* = 0.001), 2PD (*p* = 0.005), numb (*p* = 0.001), and Phalen (*p* = 0.001); Pain was not significantly different (*p* = 0.143) (Table 2).

### 3.2. Multiple Regression Analysis

Background factors of each assessment that were significantly different in Result 1 were analyzed using multiple regression analysis. These results of multiple regression analysis showed significant differences in grip-s (F = 1.120, *p* < 0.366, R = 0.437, and R^2^ = 0.192), and pinch-s (F = 1.513, *p* = 0.155, R = 0.492, and R^2^ = 0.242) for gender and SCV. SWMT (F = 2.18, *p* < 0.03) in the start-treat and OT-inter. 2PD (F = 1.14, *p* < 0.35) in Start-treat. Pain (F = 1.77, *p* = 0.08), numb (F = 1.77, *p* = 0.08), and Phalen (F = 1.32, *p* = 0.24) were not affected by independent variables set in this study (Table 3, Table 4, Table 5, Table 6, Table 7, Table 8 and Table 9).

## 4. Discussion

### 4.1. Effectiveness of Surgical Treatment Combined with NGE

In this study, a significant treatment effect was observed for grip-s, pinch-s, SWMT, 2PD, numb, and Phalen, but no effect for pain. The pathophysiology of CTS is a combination of mechanical and ischemic injury of the median nerve in the carpal tunnel [39]. This type of strangulation neuropathy, CTS, can be greatly improved by surgically releasing the pressure on the carpal tunnel. However, the effectiveness of symptom improvement greatly varies depending on the postoperative care method [30,31,32]. Degnan et al. (1997) reported that early control of edema is important after surgery for CTS [30]. Cook et al. (1995) reported that splint immobilization of the wrist joint postoperatively causes deformity, therefore, early exercise of the fingers and wrist joint is important [31]. Steyers et al. (2002) reported that mobilization should be performed early postoperatively to promote gliding of tendons and nerves [32]. Effects of early mobilization are expected to include nerve stimulation, promotion of venous return, edema resolution and prevention, and reduction of carpal tunnel pressure [10,33,38]. The study results showed that CTS treatment with surgery and NGE can improve hand function, and this finding is in agreement with those of previous studies. Additionally, the positive angle of the Phalen test may have been affected.

Conversely, the pain was not affected by the combination of surgery and NGE as a background factor. This result may be because we did not specify the type or cause of pain evaluated in this study.

### 4.2. Functions and Factors Improved by Surgery and NGE

Surgical treatment combined with NGE significantly improved grip-s, Pinch-s, SWMT, 2PD, Numb, and Phalen. Regression analysis revealed the possible factors associated with the improvement of these parameters. Grip-s and pinch-s were affected by gender and SCV, SWMT by start-treat and OT-inter, and 2PD by start-treat and OT-inter. Pain, numbness and Phalen are the factors without effect on these. Results of this study showed that SCV and gender affected grip-s and pinch-s. SWMT and 2PD were influenced by start-treat. In contrast, numb and Phalen were not affected by factors as independent variables. Furthermore, SCV and organic factors such as gender were involved in the background of intervention effects in grip-sand pinch-s. Werner et al. (2011) reported that in mild CTS, SCV was delayed but MCV was normal; when CTS was moderate, MCV was delayed in addition to SCV [41]. In other words, SCV was impaired earlier than MCV, and myelin lesion was impaired earlier than the axon lesion. Lew et al. reported that SCV is a sensitive test for CTS [42]. The results of this study also suggest that the involvement of SCV in muscle strength, such as grip-s and pinch-s, sensitively reflects the degree of disability before the MCV effect is achieved.

In both SWMT and 2PD results, start-treat was involved. Gelberman et al. (1981) reported that delayed surgery for CTS could damage the median nerve [43]. Additionally, Choi et al. and Townshend et al. reported that prolonged and severe compression of the median nerve can cause axonal degeneration, rendering SCV immeasurable [44,45]. In other words, we thought that the duration of compression and damage to the median nerve would be a background factor behind the improved SWMT and 2PD.

Of note, pain could not be explained by any factor. Neuropathic pain is a very important target of treatment, but pain improvement is difficult because of the wide variety of causes, not just in CTS [46]. Therefore, to address pain caused by CTS, we should perform a further study, such as a study to explore the cause of pain in patients with CTS.

### 4.3. Limitation

In this study, the combination of surgery and NGE improved the treatment for muscle strength and sensation, and background factors were identified. However, background factors of numb and Phalen were not identified, which had an improved effect in variables considered as factors, and pain. The present study is limited in its pain examination due to the various possible causes for the pain mechanism.

There was another concern about the interpretation of our data. We did not collect affectable parameters that affect the results, including smoking [47] or alcohol habits [48], physical sporting performance [49], and kinesiophobia [50]. In interpreting the results of this study, it should be noted that the influence of these factors has not been considered. A further study including these parameters is needed.

### 4.4. Clinical Implications

The results of our current study indicate the positive effect of combination of surgery for CTS and NGE on motor and sensory function in the affected hand, as in previous studies [10,11,12,22,23]. Therefore, occupational or physical therapy can be recommended as after-therapy to improve motor and sensory function. However, for pain, our result indicates that this combination therapy cannot be enough. If pain remains a crucial problem, other treatments need to be considered.

## 5. Conclusions

Treatment with NGE after CTS surgery increased the treatment effect on grip-s, pinch-s, SWMT, 2PD, numbness, and Phalen-positive angle. Furthermore, SCV was a background factor for muscle strength, and time-to-treatment initiation was a background factor for sensation.

## Figures and Tables

**Table 1:** Attributes.

	*n*
Subjects	67
Hands (Rt/Lt)	86 (48/38)
Age (mean ± SD)	66.8 ± 14.1
Gender	M: 24; F: 43
Dialysis	4
Trigger finger	20
TEA ^1^ (Rt/Lt)	43 (26/17)

^1^ TEA: thenar eminence atrophy; M: Male, F: Female.

**Table 2:** Comparison between preoperative and final assessments.

	Difference between Pre vs. Post		95% CL		*p*-Value	
	Average	Standard Error	Lower	Upper		
Grip-s (kgf)	1.13	4.45	0.05	2.21	0.04	**
Pinch-s (kgf)	0.45	1.33	0.13	0.77	0.007	**
SWMT	0.96	1.26	0.66	1.26	0.001	**
2PD (mm)	1.71	4.99	0.52	2.91	0.005	**
Numb (score)	4.18	2.38	3.60	4.75	0.001	**
Pain (score)	0.56	3.19	−0.20	1.33	0.14	
Phalen (angle)	16.01	19.49	11.37	20.66	0.001	**

** *p* < 0.01.

**Table 3:** Independent variable for grip-s.

	B	Standard Error	β	B (95% CI)		F	t	*p*-Value		VIF
				Lower	Upper					
Age	0.03	0.09	0.05	−0.14	0.20	0.12	0.35	0.727		1.43
Gender	−6.03	2.53	−0.33	−11.10	−0.96	5.70	−2.39	0.021	*	1.20
Fault side	2.42	2.36	0.14	−2.33	7.16	1.05	1.02	0.311		1.21
Bilateral	0.55	2.57	0.03	−4.61	5.70	0.05	0.21	0.832		1.28
Trigger finger	−2.19	1.98	−0.14	−6.17	1.78	1.22	−1.11	0.274		1.09
Dialysis	−4.64	5.54	−0.12	−15.76	6.47	0.70	−0.84	0.406		1.23
TEA	−0.34	2.43	−0.02	−5.23	4.54	0.02	−0.14	0.888		1.33
MCV	−0.08	0.14	−0.08	−0.36	0.20	0.34	−0.58	0.562		1.24
SCV	0.24	0.12	0.30	0.00	0.48	4.11	2.03	0.048	*	1.38
Start-treat	0.04	0.03	0.18	−0.02	0.09	1.83	1.35	0.182		1.15
OT-inter	−0.01	0.10	−0.02	−0.21	0.19	0.01	−0.11	0.914		1.23
Constant	−0.05	13.03		−26.20	26.10	0.00	0.00	0.997		

B: partial regression coefficient; β: standardized partial regression coefficient. 95% CI: 95% confidence interval; VIF: Variance inflation factor; * *p* < 0.05.

**Table 4:** Independent variable for pinch-s.

	B	Standard Error	β	B (95% CI)		F	t	*p*-Value		VIF
				Lower	Upper					
Age	0.00	0.02	−0.03	−0.04	0.03	0.05	−0.23	0.822		1.43
Gender	−1.40	0.48	−0.39	−2.36	−0.44	8.54	−2.92	0.005	**	1.20
Fault side	0.25	0.45	0.07	−0.65	1.14	0.30	0.55	0.587		1.21
Bilateral	0.24	0.49	0.07	−0.74	1.21	0.24	0.49	0.629		1.28
Trigger finger	−0.20	0.38	−0.07	−0.96	0.55	0.29	−0.54	0.594		1.09
Dialysis	−0.77	1.05	−0.10	−2.87	1.34	0.53	−0.73	0.468		1.23
TEA	−0.44	0.46	−0.13	−1.37	0.48	0.92	−0.96	0.343		1.33
MCV	−0.02	0.03	−0.11	−0.07	0.03	0.64	−0.80	0.428		1.24
SCV	0.05	0.02	0.29	0.00	0.09	4.18	2.04	0.046	*	1.38
Start-treat	0.01	0.01	0.21	0.00	0.02	2.65	1.63	0.109		1.15
OT-inter	−0.02	0.02	−0.15	−0.06	0.02	1.22	−1.10	0.275		1.23
Constant	2.18	2.47		−2.78	7.13	0.78	0.88	0.383		

B: partial regression coefficient; β: standardized partial regression coefficient. 95% CI: 95% confidence interval; VIF: Variance inflation factor; * *p* < 0.05, ** *p* < 0.01.

**Table 5:** Independent variable for SWMT.

	B	Standard Error	β	B (95% CI)		F	t	*p*-Value	VIF	
				Lower	Upper					
Age	−0.01	0.02	−0.06	−0.04	0.02	0.22	−0.47	0.640		1.43
Gender	−0.62	0.44	−0.18	−1.50	0.26	1.99	−1.41	0.164		1.20
Fault side	0.45	0.41	0.14	−0.38	1.27	1.18	1.08	0.283		1.21
Bilateral	−0.25	0.45	−0.07	−1.15	0.64	0.32	−0.56	0.576		1.28
Trigger finger	0.07	0.34	0.03	−0.62	0.76	0.05	0.21	0.831		1.09
Dialysis	−0.19	0.96	−0.03	−2.12	1.73	0.04	−0.20	0.840		1.23
TEA	0.20	0.42	0.06	−0.65	1.05	0.22	0.47	0.639		1.33
MCV	0.03	0.02	0.14	−0.02	0.08	1.28	1.13	0.263		1.24
SCV	0.03	0.02	0.19	−0.01	0.07	2.03	1.42	0.161		1.38
Start-treat	0.01	0.00	0.30	0.00	0.02	5.81	2.41	0.019	*	1.15
OT-inter	−0.05	0.02	−0.33	−0.08	−0.01	6.82	−2.61	0.012	*	1.23
Constant	−1.46	2.26		−6.00	3.09	0.41	−0.64	0.523		

B: partial regression coefficient; β: standardized partial regression coefficient. 95% CI: 95% confidence interval; VIF: Variance inflation factor; * *p* < 0.05.

**Table 6:** Independent variable for 2PD.

	B	Standard Error	β	B (95% CI)		F	t	*p*-Value		VIF
				Lower	Upper					
Age	0.02	0.05	0.08	−0.07	0.12	0.26	0.51	0.611		1.43
Gender	−0.92	1.38	−0.09	−3.69	1.86	0.44	−0.66	0.509		1.20
Fault side	1.55	1.29	0.16	−1.04	4.15	1.44	1.20	0.236		1.21
Bilateral	1.82	1.41	0.18	−1.00	4.65	1.68	1.30	0.201		1.28
Trigger finger	0.55	1.09	0.07	−1.63	2.73	0.26	0.51	0.616		1.09
Dialysis	−1.54	3.03	−0.07	−7.62	4.55	0.26	−0.51	0.614		1.23
TEA	−0.75	1.33	−0.08	−3.43	1.92	0.32	−0.57	0.574		1.33
MCV	0.10	0.08	0.18	−0.05	0.25	1.68	1.30	0.201		1.24
SCV	0.08	0.06	0.18	−0.05	0.21	1.52	1.23	0.224		1.38
Start-treat	0.03	0.01	0.27	0.00	0.06	4.23	2.06	0.045	*	1.15
OT-intere	0.03	0.05	0.07	−0.08	0.14	0.25	0.50	0.618		1.23
Constant	−16.28	7.14		−30.60	−1.96	5.20	−2.28	0.027	*	

B: partial regression coefficient; β: standardized partial regression coefficient. 95% CI: 95% confidence interval; VIF: Variance inflation factor; * *p* < 0.05.

**Table 7:** Independent variable for pain.

	B	Standard Error	β	B (95% CI)		F	t	*p*-Value		VIF
				Lower	Upper					
Age	0.01	0.03	0.05	−0.05	0.07	0.21	0.35	0.73		1.43
Gender	−1.80	0.89	−0.26	−3.59	−0.01	4.07	−2.02	0.07		1.20
Fault side	1.31	0.84	0.20	−0.36	2.99	2.47	1.57	0.12		1.21
Bilateral	−0.24	0.91	−0.04	−2.07	1.58	0.07	−0.27	0.79		1.28
Trigger finger	−0.02	0.70	0.00	−1.43	1.39	0.00	−0.03	0.98		1.09
Dialysis	−0.81	1.96	−0.05	−4.74	3.12	0.17	−0.42	0.68		1.23
TEA	1.62	0.86	0.26	−0.11	3.35	3.55	1.88	0.07		1.33
MCV	−0.02	0.05	−0.05	−0.12	0.08	0.16	−0.40	0.69		1.24
SCV	0.07	0.04	0.24	−0.01	0.16	3.14	1.77	0.08		1.38
Start-treat	0.02	0.01	0.22	0.00	0.04	3.07	1.75	0.09		1.15
OT-intere	−0.06	0.04	−0.21	−0.13	0.01	2.51	−1.58	0.12		1.23
Constant	−1.76	4.61		−11.01	7.49	0.15	−0.38	0.70		

B: partial regression coefficient; β: standardized partial regression coefficient. 95% CI: 95% confidence interval; VIF: Variance inflation factor.

**Table 8:** Independent variable for numb.

	B	Standard Error	β	B (95% CI)		F	t	*p*-Value		VIF
				Lower	Upper					
Age	−0.03	0.03	−0.12	−0.09	0.04	0.71	−0.84	0.404		1.43
Gender	0.21	0.90	0.03	−1.59	2.02	0.06	0.24	0.812		1.20
Fault side	0.77	0.84	0.12	−0.92	2.46	0.84	0.92	0.363		1.21
Bilateral	−0.87	0.91	−0.13	−2.71	0.96	0.91	−0.96	0.343		1.28
Trigger finger	0.85	0.70	0.15	−0.56	2.27	1.47	1.21	0.232		1.09
Dialysis	−0.07	1.97	0.00	−4.02	3.88	0.00	−0.03	0.973		1.23
TEA	−0.08	0.87	−0.01	−1.82	1.65	0.01	−0.09	0.926		1.33
MCV	0.08	0.05	0.21	−0.02	0.18	2.47	1.57	0.122		1.24
SCV	0.08	0.04	0.27	0.00	0.17	3.83	1.96	0.056		1.38
Start-treat	0.01	0.01	0.18	−0.01	0.03	2.12	1.45	0.152		1.15
OT-intere	−0.03	0.04	−0.11	−0.10	0.04	0.72	−0.85	0.399		1.23
Constant	−9.51	4.63		−18.81	−0.22	4.22	−2.05	0.045	*	

B: partial regression coefficient; β: standardized partial regression coefficient. 95% CI: 95% confidence interval; VIF: Variance inflation factor; * *p* < 0.05.

**Table 9:** Independent variable for Phalen.

	B	Standard Error	β	B (95% CI)		F	t	*p*-Value		VIF
				Lower	Upper					
Age	0.39	0.24	0.24	−0.09	0.88	2.64	1.63	0.110		1.43
Gender	−3.52	7.07	−0.07	−17.71	10.66	0.25	−0.50	0.620		1.20
Fault side	5.10	6.62	0.10	−8.18	18.38	0.59	0.77	0.444		1.21
Bilateral	7.56	7.19	0.15	−6.87	21.99	1.11	1.05	0.298		1.28
Trigger finger	−7.77	5.55	−0.18	−18.90	3.37	1.96	−1.40	0.168		1.09
Dialysis	2.62	15.50	0.02	−28.49	33.72	0.03	0.17	0.867		1.23
TEA	−7.04	6.81	−0.15	−20.72	6.63	1.07	−1.03	0.306		1.33
MCV	0.11	0.39	0.04	−0.68	0.90	0.08	0.28	0.781		1.24
SCV	0.42	0.33	0.18	−0.24	1.09	1.63	1.28	0.207		1.38
Start-treat	0.13	0.08	0.22	−0.03	0.28	2.76	1.66	0.102		1.15
OT-intere	−0.33	0.28	−0.16	−0.88	0.23	1.37	−1.17	0.247		1.23
Constant	−36.69	36.48		−109.88	36.51	1.01	−1.01	0.319		

B: partial regression coefficient; β: standardized partial regression coefficient. 95% CI: 95% confidence interval; VIF: Variance inflation factor.

## Data Availability

The data presented in this study are openly available in Mendeley Data at doi:10.17632/dpyg8dw49n.2, reference number [51].
